# Aging-Related Gene-Based Prognostic Model for Lung Adenocarcinoma: Insights into Tumor Microenvironment and Therapeutic Implications

**DOI:** 10.3390/ijms252413572

**Published:** 2024-12-18

**Authors:** Jin Wang, Hailong Zhang, Yaohui Feng, Xian Gong, Xiangrong Song, Meidan Wei, Yaoyu Hu, Jianxiang Li

**Affiliations:** Department of Toxicology, School of Public Health, Suzhou Medicine College of Soochow University, Suzhou 215123, China; jin.wang93@outlook.com (J.W.); 20224247032@stu.suda.edu.cn (H.Z.); fyh134679shangan@163.com (Y.F.); 20224247016@stu.suda.edu.cn (X.G.); 20234247003@stu.suda.edu.cn (X.S.); 20234247035@stu.suda.edu.cn (M.W.); 15221856821@163.com (Y.H.)

**Keywords:** aging, lung adenocarcinoma, prognostic model, cellular senescence, XRCC6

## Abstract

Lung cancer remains the leading cause of cancer-related mortality globally, with a poor prognosis primarily due to late diagnosis and limited treatment options. This research highlights the critical demand for advanced prognostic tools by creating a model centered on aging-related genes (ARGs) to improve prediction and treatment strategies for lung adenocarcinoma (LUAD). By leveraging datasets from The Cancer Genome Atlas (TCGA) and Gene Expression Omnibus (GEO), we developed a prognostic model that integrates 14 ARGs using the least absolute shrinkage and selection operator (LASSO) alongside Cox regression analyses. The model exhibited strong predictive performance, achieving area under the curve (AUC) values greater than 0.8 for one-year survival in both internal and external validation cohorts. The risk scores generated by our model were significantly correlated with critical features of the tumor microenvironment, including the presence of cancer-associated fibroblasts (CAFs) and markers of immune evasion, such as T-cell dysfunction and exclusion. Higher risk scores correlated with a more tumor-promoting microenvironment and increased immune suppression, highlighting the model’s relevance in understanding LUAD progression. Additionally, XRCC6, a protein involved in DNA repair and cellular senescence, was found to be upregulated in LUAD. Functional assays demonstrated that the knockdown of XRCC6 led to decreased cell proliferation, whereas its overexpression alleviated DNA damage, highlighting its significance in tumor biology and its potential therapeutic applications. This study provides a novel ARG-based prognostic model for LUAD, offering valuable insights into tumor dynamics and the tumor microenvironment, which may guide the development of targeted therapies and improve patient outcomes.

## 1. Introduction

Globally, the incidence and mortality rates of cancer are on the rise. Among all types of cancer, lung cancer is the most common, accounting for 11.6% of all cases. Lung cancer continues to be the foremost cause of cancer-related mortality worldwide, accounting for 18.4% of all cancer mortality and placing substantial social and economic burdens on society [1,2]. The prognosis for lung cancer is difficult due to the absence of early diagnostic tools and the delayed onset of symptoms during disease progression, which restricts treatment options and reduces survival rates [3]. The World Health Organization (WHO) classifies lung tumors into two primary types: non-small cell lung cancer (NSCLC), which comprises 80–85% of all lung cancer cases, and small cell lung cancer (SCLC), which accounts for the remaining 15% [4,5]. NSCLC can be further subdivided into lung adenocarcinoma (LUAD), lung squamous cell carcinoma (LUSC), and large cell carcinoma (LCC).

With the increase in the elderly population, a deeper understanding of the biological underpinnings of aging is urgently needed. Aging is a major risk factor for all age-related chronic diseases in adults, resulting in changes to lung function, remodeling of lung architecture, a decrease in regenerative capacity, and increased vulnerability to both acute and chronic pulmonary diseases [6,7]. Aging is characterized by a constellation of distinct yet interrelated biological alterations, which include genomic instability, telomere erosion, epigenetic modifications, proteostasis impairment, compromised macroautophagy, perturbed nutrient-sensing pathways, mitochondrial dysregulation, the onset of cellular senescence, depletion of stem cell reserves, disruption of intercellular signaling, pervasive chronic inflammation, and microbial dysbiosis [8]. These characteristics are similar to the hallmarks of cancer, especially genetic ability and epigenetic modifications [9]. Senescent cells accumulate with age in various tissues and are implicated in a range of age-related diseases, including cancer [6]. Cellular senescence is viewed as a fundamental characteristic of cancer [10].

The Aging Atlas is a multi-omics database focused on aging biology that collects aging-related genes (ARGs) from existing literature [11]. These ARGs were applied to build a prognostic model utilizing a LUAD dataset from TCGA and an additional dataset from GEO. The model was built employing the least absolute shrinkage and selection operator (LASSO) alongside Cox regression analysis. The clinical and biological relevance of the risk factors derived from the model was further assessed using bioinformatics techniques.

## 2. Results

### 2.1. Datasets

Two LUAD patient cohorts and their clinical data were obtained from the TCGA and GEO databases. Table 1 and Table S1 outline the demographic and clinical characteristics of the training and internal testing sets. After excluding samples lacking complete clinical data from the TCGA-LUAD dataset, this study focused on 504 LUAD patients, with 183 alive and 321 deceased, by the conclusion of the follow-up period (median follow-up: 1.789 years). The dataset was randomly split into a training set (*n* = 303) and an internal testing set (*n* = 201). Consistent with expectations, there were no significant differences in the key clinicopathological features among the training set, testing set, and the overall TCGA-LUAD dataset (Table 1). Furthermore, this study incorporated the GEO dataset GSE31210, which consisted of 226 LUAD patients, of whom 37.81% had died by the end of the follow-up period (median follow-up duration: 4.720 years).

### 2.2. Prognostic Modeling

**Figure 1:**
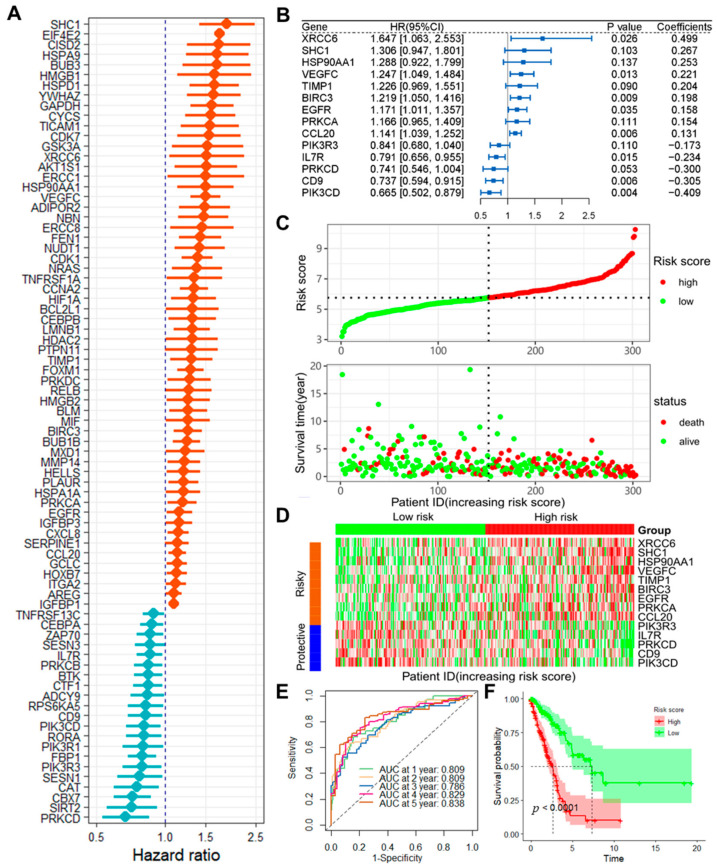
Construction and performance of the ARGs prognostic model. (**A**) Identification of prognostic ARGs via univariate COX analysis; (**B**) Development of the prognostic model through multivariate COX analysis; (**C**) Analysis of risk score distributions and survival outcomes across high-risk and low-risk groups in the training cohort; (**D**) Heatmap illustrating the expression levels of ARGs in high-risk and low-risk groups within the training cohort; (**E**) TimeROC curves displaying ROC curves and AUC values for 1–5 years in the training cohort; (**F**) Survival curves for high- and low-risk groups in the training cohort.

**Figure 2:**
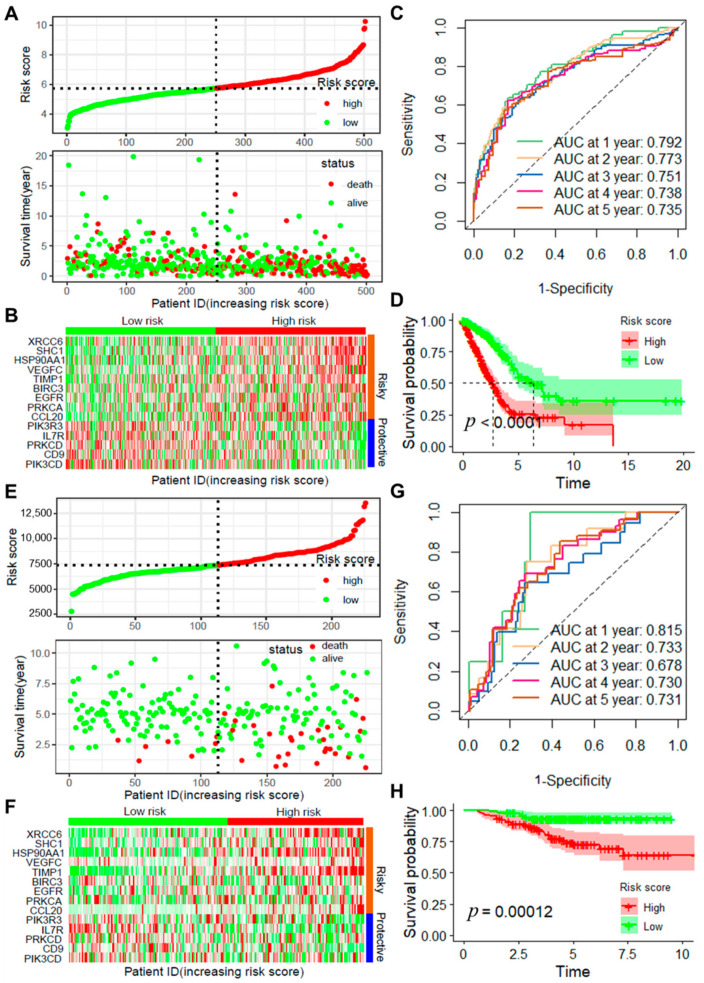
Validation of the model’s robustness in TCGA_LUAD and GSE31210 datasets. (**A**) Distribution of risk scores and survival outcomes in high- and low-risk groups within the TCGA_LUAD dataset; (**B**) Heatmap showing the expression of ARGs in high- and low-risk groups within the TCGA_LUAD dataset; (**C**) TimeROC curves displaying ROC curves and AUC values for 1–5 years in the TCGA_LUAD dataset; (**D**) Survival curves for high- and low-risk groups in the TCGA_LUAD dataset; (**E**) Distribution of risk scores and survival outcomes in high- and low-risk groups within the GSE31210 dataset; (**F**) Heatmap showing the expression of ARGs in high- and low-risk groups within the GSE31210 dataset; (**G**) TimeROC curves displaying ROC curves and AUC values for 1–5 years in the GSE31210 dataset; (**H**) Survival curves for high- and low-risk groups in the GSE31210 dataset.

A univariate Cox analysis identified 79 prognostic ARGs (Figure 1A). Subsequent gene selection through LASSO regression yielded 23 genes (Figure S1). These selected genes were then analyzed using a stepwise multivariate Cox regression, and the outcomes are presented in Figure 2B. The final risk model derived from this study is as follows: Risk score = CD9 _Exp_ × (−0.305) + BIRC3 _Exp_ × (0.198) + HSP90AA1 _Exp_ × (0.253) + TIMP1 _Exp_ × (0.204) + CCL20 _Exp_ × (0.131) + PIK3R3 _Exp_ × (−0.173) + EGFR _Exp_ × (0.158) + VEGFC _Exp_ × (0.221) + PRKCA _Exp_ × (0.154) + SHC1 _Exp_ × (0.267) + PRKCD _Exp_ × (−0.300) + IL7R _Exp_ × (−0.234) + PIK3CD _Exp_ × (−0.409) + XRCC6 _Exp_ × (0.499). In the training cohort, individuals with elevated risk scores experienced reduced overall survival times and increased mortality rates (Figure 1C and Figure S2). The heatmap demonstrates that the risk genes identified in the model are significantly upregulated in high-risk samples, while protective genes are notably expressed at higher levels in low-risk samples (Figure 1D). The ROC curve indicates that the model exhibits strong predictive capability, with AUC values exceeding 0.8 for the 1, 2, 4, and 5-year time frames (Figure 1E), and shows that high-risk patients have a significantly worse prognosis compared to those in the low-risk group (Figure 1F).

### 2.3. Model Validation

To evaluate the reliability and robustness of the model, risk scores were determined for all samples in the internal test cohort, the entire TCGA-LUAD dataset, and the external validation cohort. In the internal test cohort, the AUCs for the 1-year and 2-year ROC curves were 0.753 and 0.714, respectively (Figure S3A), and the survival curves indicated better prognosis for low-risk patients compared to high-risk patients (Figure S3B). Further analysis of the prognostic model’s performance in the entire TCGA-LUAD dataset revealed the distribution of survival times and outcomes for high- and low-risk groups (Figure 2A,B), along with the differential expression of ARGs in all samples. Additionally, the AUC values for the ROC curves at 1 to 3 years exceeded 0.75 (Figure 2C), and the survival curves indicated a significantly more favorable prognosis for the low-risk group in comparison to the high-risk group (Figure 2D). The model’s reliability was further validated in another independent lung adenocarcinoma dataset, yielding results consistent with the TCGA-LUAD dataset (Figure 2E–H).

### 2.4. Risk Score as an Independent Prognostic Factor

**Figure 3:**
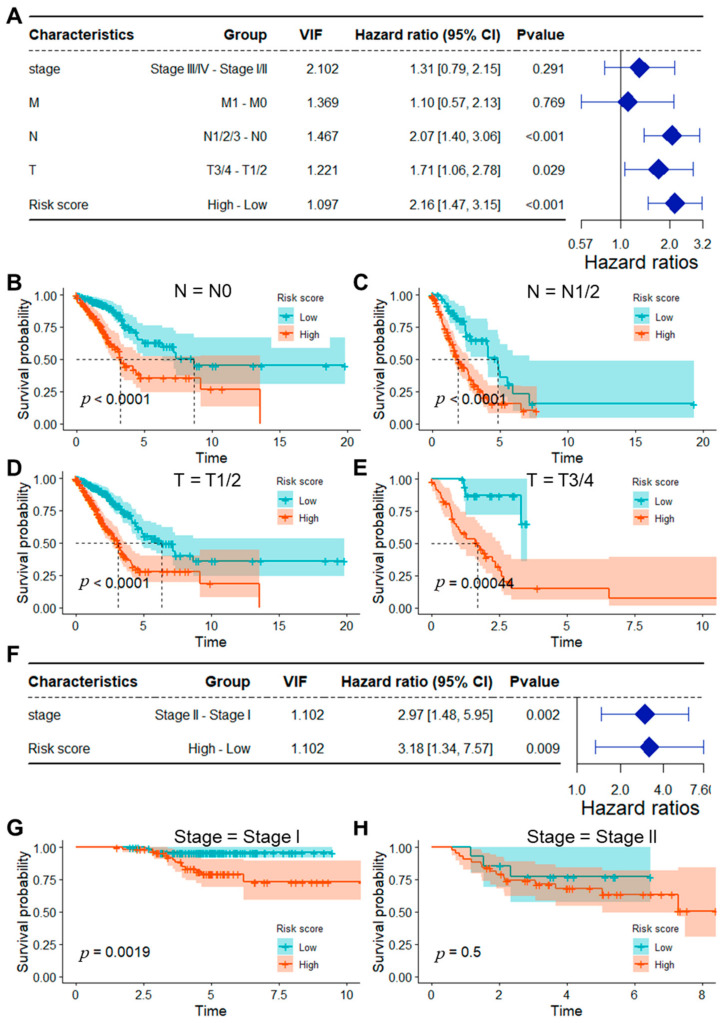
Identification of risk score as an independent prognostic indicator for overall survival in lung adenocarcinoma. (**A**) Outcomes of multivariate COX analysis that integrated significant factors identified in the univariate COX analysis from the TCGA_LUAD dataset; (**B**,**C**) Survival curves for high-risk and low-risk groups classified by N stage within the TCGA_LUAD dataset; (**D**,**E**) Survival curves for high-risk and low-risk groups categorized by T stage in the TCGA_LUAD dataset; (**F**) Findings from multivariate COX analysis that included significant factors from the univariate COX analysis in the GSE31210 dataset; (**G**,**H**) Survival curves for high- and low-risk groups stratified by Stage in the GSE31210 dataset. VIF, variance inflation factor.

Firstly, the risk scores and other clinicopathological features from the TCGA_LUAD dataset were incorporated into a univariate Cox analysis. Subsequently, significant variables, including distant metastasis (M), regional lymph node (N), primary tumor (T), stage, and risk score, were subjected to multivariate Cox analysis (Figure S4A). The results revealed that N stage, T stage, and risk score were independent prognostic indicators for lung adenocarcinoma (Figure 3A). To further validate these findings, we performed a stratification analysis based on N and T stages. This analysis revealed that high-risk patients exhibited a significantly poorer prognosis across all N and T stages, supporting the robustness of the risk score as a prognostic factor (Figure 3B–E). Similarly, in the GSE31210 dataset, univariate Cox analysis identified stage and risk score as significant prognostic variables (Figure S4B). The subsequent multivariate Cox analysis confirmed that both stage and risk score were independent prognostic factors for survival in this cohort (Figure 3F). Stratification by stage further demonstrated that high-risk patients had markedly worse prognoses across different stages of the disease (Figure 3G,H). These modifications to the figures, including the inclusion of additional quantitative data and clearer visual representation, were made to better highlight the prognostic significance of these factors and provide a more compelling demonstration of their independent predictive value.

### 2.5. Nomogram Based on Risk Score Shows Superior Predictive Performance

**Figure 4:**
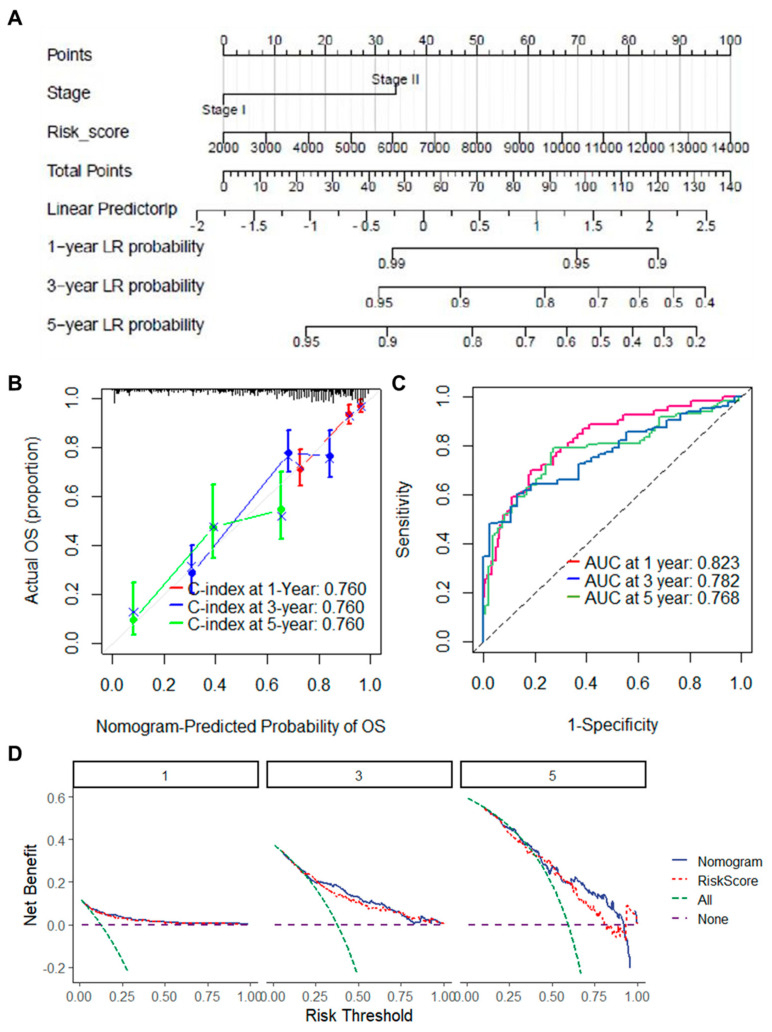
Construction and validation of the clinical predictive nomogram based on the TCGA_LUAD dataset. (**A**) A clinical predictive nomogram that integrates independent prognostic factors identified via multivariate COX regression analysis, where the total points displayed on the lower scale indicate the probabilities of overall survival at 1, 3, and 5 years; (**B**) Calibration plots illustrating the 1-, 3-, and 5-year overall survival predictions derived from the nomogram, using the GSE31210 dataset; (**C**) Kaplan–Meier curves for overall survival based on nomogram scores in the GSE31210 dataset; (**D**) Decision curve analysis (DCA) curves for the nomogram and risk score, predicting overall survival at 1, 3, and 5 years in the GSE31210 dataset. ARGs refer to aging-related genes; OS denotes overall survival; DCA stands for decision curve analysis.

To conduct a more comprehensive assessment of the clinical relevance of the ARGs prognostic model, we developed clinical predictive nomograms based on the independent prognostic factors identified from multivariate Cox regression analysis of the TCGA_LUAD and GSE31210 datasets (Figure 4A and Figure S5A). To enhance the robustness of our results, internal validation was performed using bootstrap C-index calculations, which yielded values of ≥0.760 in both the TCGA_LUAD and GSE31210 datasets, demonstrating the strong predictive performance of the model. Additionally, calibration plots were generated to assess the agreement between predicted and observed outcomes, further supporting the reliability of the nomogram (Figure 4B and Figure S5B). The ROC curves further substantiated the high predictive accuracy of the nomogram-based risk scores for overall survival. The AUC was 0.823 for the TCGA_LUAD dataset and 0.913 for the GSE31210 dataset at the one-year time point, highlighting the excellent discriminative ability of the model (Figure 4C and Figure S5C). To evaluate the clinical utility of the nomogram, decision curve analysis (DCA) was performed, showing that the nomogram provides greater net benefit compared to both no intervention and treating all patients across a wide range of threshold probabilities (Figure 4D and Figure S5D). Importantly, the nomogram demonstrated better net benefit than either treating all patients or not treating any patients and outperformed the risk score alone, emphasizing its superior clinical applicability.

### 2.6. Risk Score Correlates with Immune Cell Infiltration

**Figure 5:**
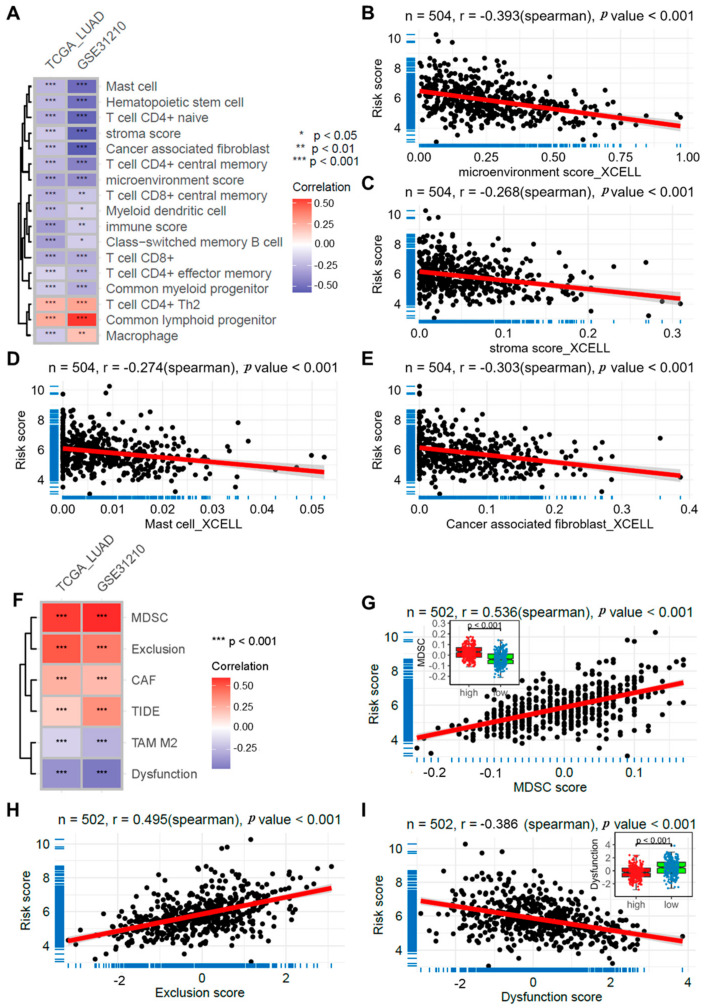
Risk Score Correlates with Immune Cell Infiltration. (**A**) A heatmap illustrating the correlation between the risk score and immune cell infiltration scores derived from XCELL in the TCGA_LUAD and GSE31210 datasets; Scatter plots depicting correlations between the risk score and the microenvironment score (**B**), stromal score (**C**), mast cell infiltration (**D**), and cancer-associated fibroblasts (CAF) (**E**) in the TCGA_LUAD dataset; (**F**) A heatmap displaying the correlation between the risk score and infiltration scores calculated by TIMER in the TCGA_LUAD and GSE31210 datasets; Scatter plots illustrating correlations between the risk score and the MDSC infiltration score (**G**), T-cell exclusion (**H**), and T-cell dysfunction (**I**) in the TCGA_LUAD dataset. TCGA, LUAD, CAF, TAM, MDSC.

Using the XCELL algorithm, immune cell infiltration scores were calculated for all samples in the TCGA-LUAD and GSE31210 datasets. Correlation analysis indicated that the risk score had a significant association with both the immune microenvironment score (Figure 5B) and the stromal score (Figure 5C), yielding correlation coefficients of −0.393 and −0.268, respectively. Additionally, the risk score was significantly correlated with various immune cell infiltration scores (Figure 5A), including mast cell (r = −0.274, Figure 5D) and cancer-associated fibroblast (CAF, r = −0.303, Figure 5E). Furthermore, the risk score was significantly associated with infiltration scores derived from the TIMER online tool, including MDSC, CAF, and TAM M2 infiltration scores, T-cell exclusion and dysfunction scores, and TIDE scores (Figure 5F–I). Specifically, the MDSC infiltration score (r = 0.536, Figure 5G) and CAF infiltration and exclusion scores (r = 0.495, Figure 5H) were positively correlated with the risk score, whereas the TAM M2 infiltration and dysfunction scores (r = −0.386, Figure 5I) were negatively correlated. Moreover, the high-risk group exhibited significantly higher MDSC infiltration, exclusion, and dysfunction scores compared to the low-risk group (Figure 5G–I).

### 2.7. Risk Score Correlates with Antitumor Drug Sensitivity and Cellular Senescence

**Figure 6:**
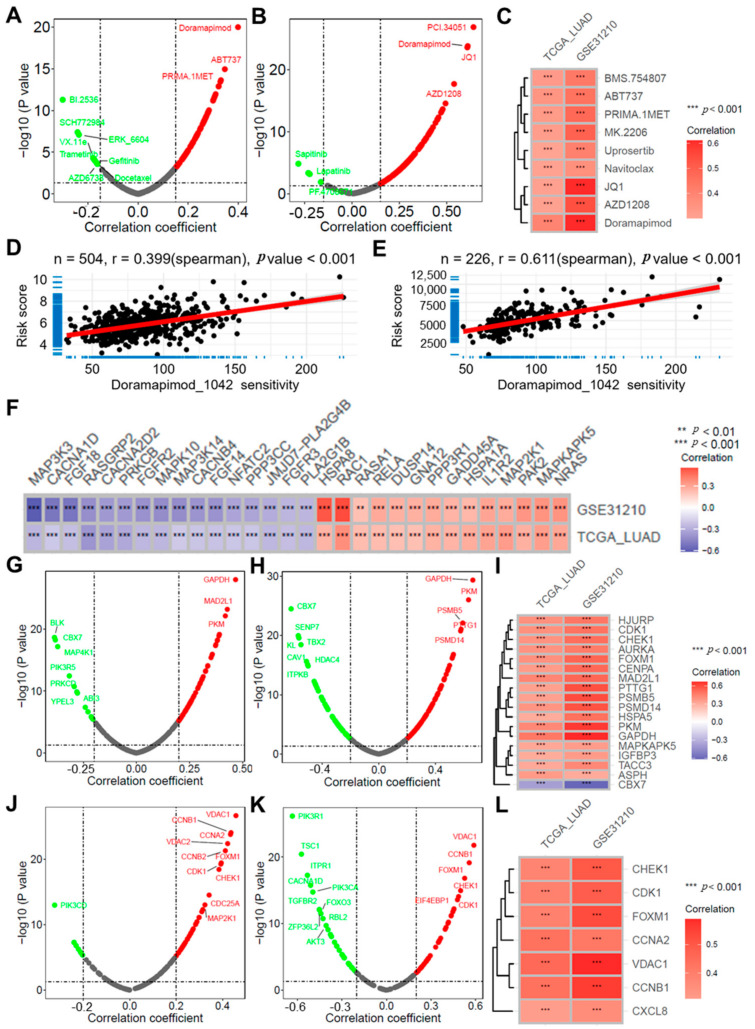
Risk Score Correlates with Antitumor Drug Sensitivity and Cellular Senescence. Volcano plots illustrating the results of the correlation analysis between the risk score and antitumor drug sensitivity in the TCGA-LUAD (**A**) and GSE31210 (**B**) datasets; (**C**) Heatmap displaying the intersection of drugs with |r| > 0.3 from both correlation results; Scatter plots depicting the relationship between the risk score and sensitivity to doramapimod in the TCGA-LUAD (**D**) and GSE31210 (**E**) datasets; (**F**) A heatmap illustrating the intersection of correlation results between the risk score and genes related to the MAPK pathway in the TCGA-LUAD and GSE31210 datasets; Volcano plots demonstrating the results of the correlation analysis between the risk score and the expression of senescence-related genes from the CellAge database in the TCGA-LUAD (**G**) and GSE31210 (**H**) datasets; (**I**) Heatmap displaying the intersection of genes with |r| > 0.3 from both correlation results; Volcano plots showing correlation analysis results between the risk score and the expression of genes in the KEGG Senescence pathway in the TCGA-LUAD (**J**) and GSE31210 (**K**) datasets; (**L**) Heatmap displaying the intersection of genes with |r| > 0.3 from both correlation results.

Antitumor drug sensitivity scores for lung adenocarcinoma samples were calculated using the Oncopredict package. Correlation analysis revealed notable associations between the risk score and sensitivity to a range of antitumor drugs in both the TCGA-LUAD and GSE31210 datasets (Figure 6A,B). As shown in Figure 6C, nine antitumor drugs with |r| > 0.3 were identified, with doramapimod exhibiting the highest correlation (r = 0.399 and 0.611, respectively, (Figure 6D,E). Doramapimod, a potent inhibitor of p38 MAPK, prompted an investigation into the relationship between the risk score and the expression levels of genes associated with the MAPK signaling pathway (Figure 6F). For cellular senescence, further correlation analysis demonstrated significant associations between the risk score and the expression of multiple genes from the CellAge database (Figure 6G–I). Additionally, genes related to the Senescence pathway from the KEGG database were analyzed, revealing significant correlations between the risk score and the expression levels of various senescence-associated genes (Figure 6J–L).

### 2.8. Risk Score Correlates with Tumor Progression

**Figure 7:**
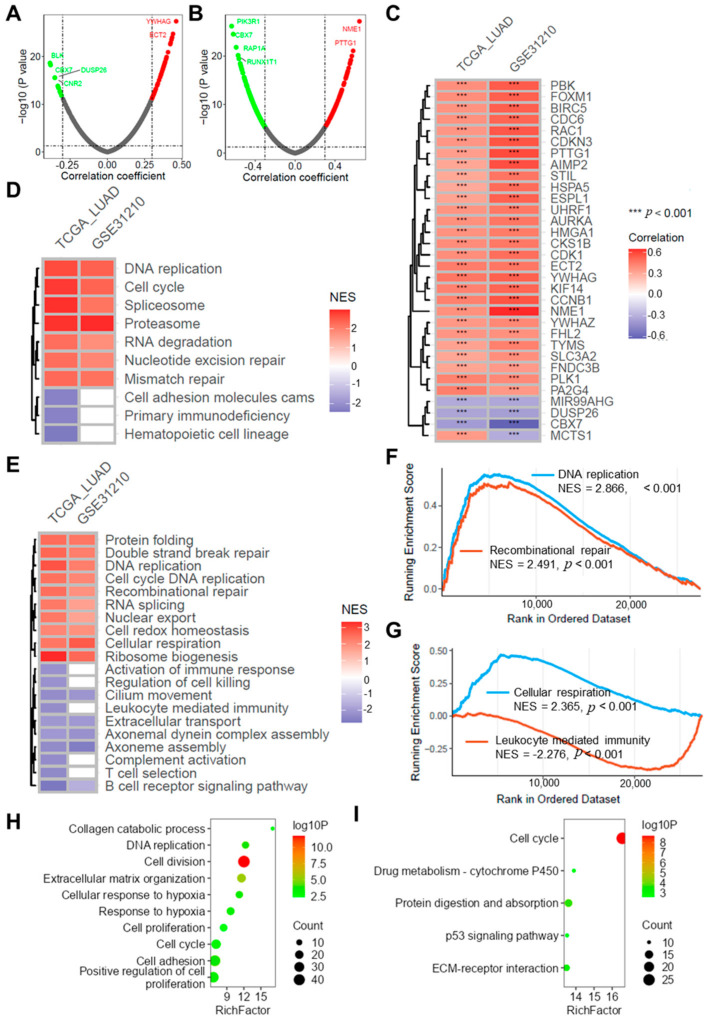
Risk score correlates with tumor progression. Volcano plots show the correlation analysis results of risk scores with oncogene expression in TCGA-LUAD (**A**) and GSE31210 (**B**) datasets. (**C**) Heatmap displaying the intersection of drugs with |r| > 0.3 from both correlation results; heatmap showing GSEA enrichment results of biological processes (**D**) and signaling pathways (**E**) in both datasets; (**F**–**G**) GSEA plots illustrating risk score associations with DNA replication, recombination repair, cellular respiration, and leukocyte mediated immunity. Bubble plots illustrate the results of enrichment analysis for biological processes (**H**) and KEGG (**I**) pathways associated with differentially expressed genes between high-risk and low-risk groups in the TCGA-LUAD dataset.

Correlation analysis indicated a significant association between the risk score and several oncogenes in both the TCGA-LUAD and GSE31210 datasets (Figure 7A–C). GSEA analysis revealed that the risk score is associated with multiple biological functions related to the tumor (Figure 7D), including DNA replication, cell cycle, nucleotide excision repair, cell adhesion molecules, and primary immunodeficiency (Figure 7D). Additionally, the risk score is linked to several tumor-associated signaling pathways (Figure 7E), such as DNA replication (NES = 2.866, Figure 7F), recombination repair (NES = 2.491, Figure 7F), cellular respiration (NES = 2.365, Figure 7G), and leukocyte-mediated immunity (NES = −2.276, Figure 7G). The differential expression analysis comparing high-risk and low-risk groups in both datasets revealed 371 upregulated genes and 379 downregulated genes (Figure S6A–D). Further enrichment analysis showed significant enrichment of these intersecting genes in multiple critical biological processes (Figure 7H) and signaling pathways (Figure 7I), including DNA replication, extracellular matrix organization, response to hypoxia, cell cycle, cell adhesion, and the p53 signaling pathway.

### 2.9. XRCC6 Is Highly Expressed in LUAD and Is Related to Cancer Progression

**Figure 8:**
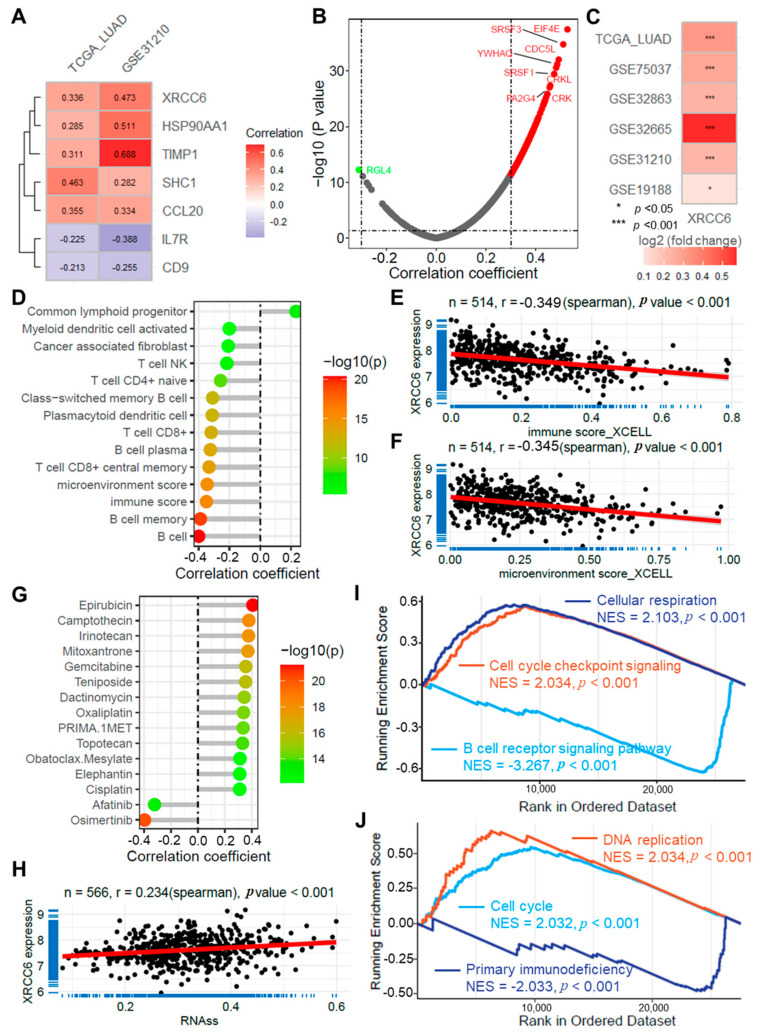
XRCC6 is highly expressed in LUAD and is related to cancer progression. (**A**) A heatmap illustrating the correlation analysis results of risk scores with ARGs in the TCGA-LUAD and GSE31210 datasets; (**B**) Volcano plot displaying correlation of *XRCC6* with cell ARGs from the CellAge database; (**C**) Heatmap displaying expression differences in *XRCC6* in tumor versus normal tissues across six LUAD datasets; (**D**) Lollipop plot showing the correlation between *XRCC6* expression and immune cell infiltration scores in the TCGA-LUAD dataset based on the XCELL algorithm; scatter plots showing correlation of *XRCC6* expression with immune score (**E**) and microenvironment score (**F**); (**G**) Lollipop plot showing correlation of XRCC6 expression with anti-cancer drug sensitivity scores in TCGA-LUAD dataset; (**H**) A scatter plot illustrating the correlation between *XRCC6* expression and tumor stemness scores as determined by the RNAss algorithm; (**I**,**J**) GSEA plots illustrating *XRCC6* associations with multiple key biological functions and signaling pathways. TCGA, LUAD.

The correlation analysis of risk scores and gene expression in the TCGA-LUAD and GSE31210 datasets identified *XRCC6* as a strongly associated gene for further validation (Figure 8A). *XRCC6* showed significant positive correlations with multiple oncogenes (r > 0.3, Figure 8B). *XRCC6* was observed to be significantly upregulated in tumor tissues from both the TCGA-LUAD and five GEO datasets (Figure 8C). Correlation analysis indicated that *XRCC6* expression is associated with various immune cell infiltrations and immune-related scores (Figure 8D), including B cells, immune score (r = −0.349, Figure 8E), and microenvironment score (r = −0.345, Figure 8F). Additionally, *XRCC6* expression correlated with sensitivity to several anti-cancer drugs (Figure 8G) and tumor stemness (Figure 8H). Further GSEA enrichment analysis revealed that *XRCC6* is related to several cancer-associated biological processes and signaling pathways, primarily including cellular respiration, cell cycle checkpoint signaling, B cell receptor signaling pathway, DNA replication, cell cycle, and primary immunodeficiency (Figure 8I,J).

### 2.10. XRCC6 Regulates Proliferation and Senescence in Lung Cancer Cells

**Figure 9:**
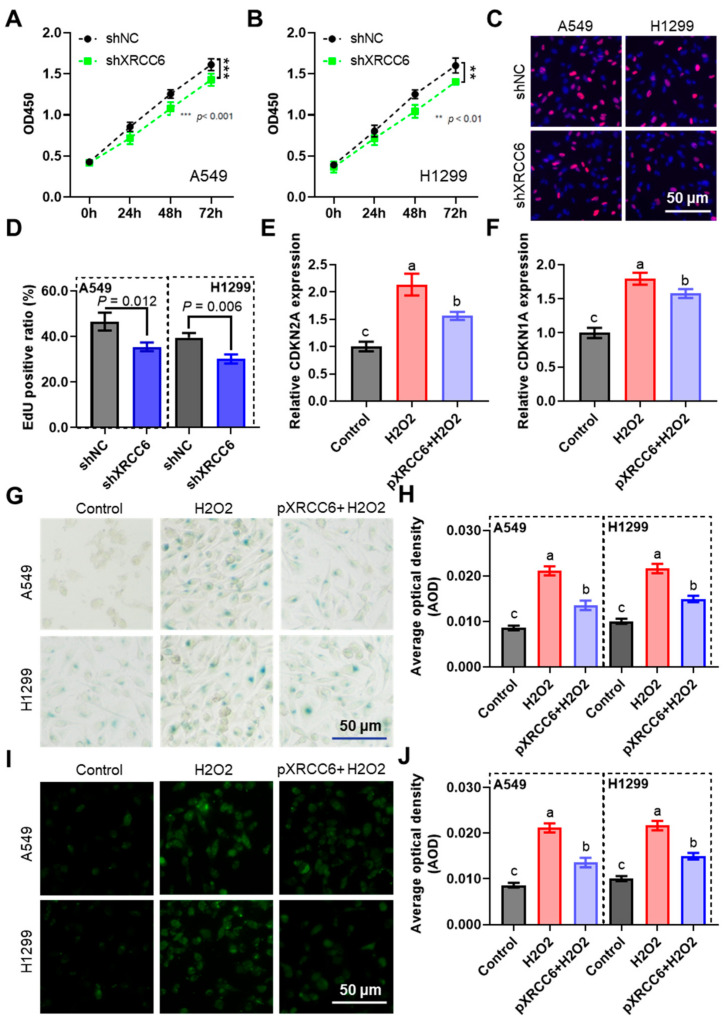
XRCC6 regulates proliferation and senescence in lung cancer cells. CCK-8 assay analyzing changes in proliferation of lung cancer cells A549 (**A**) and H1299 (**B**) following *XRCC6* knockdown; representative images (**C**) and quantitative data (**D**) of EdU assay analyzing cell proliferation; changes in *CDKN1A* (**E**) and *CDKN2A* (**F**) mRNA expression following H2O2-induced senescence and *XRCC6* overexpression; representative images (**G**) and quantification (**H**) of β-galactosidase staining; and representative images (**I**) and quantification (**J**) of γ-H2AX immunofluorescence. The different letters (a, b, c and d) represent statistically significant group differences (*p* < 0.05).

To validate the function of *XRCC6*, we constructed knockdown shRNA and overexpression plasmids for *XRCC6* (Figure S7). CCK-8 and EdU cell proliferation assays indicated that the knockdown of *XRCC6* markedly reduced the proliferation of lung cancer cells A549 and H1299 (Figure 9A–D). For cell senescence, we used 100 μM H2O2 treatment for 24 h to establish an oxidative damage-induced senescence model. The findings established that the mRNA expressions of *CDKN2A* and *CDKN1A* were significantly elevated in lung cancer cells treated with H2O2 (Figure 9E,F), and β-galactosidase staining was more intense (Figure 9G,H). Furthermore, γ-H2AX immunofluorescence results indicated that H_2_O_2_ treatment induced DNA double-strand break damage (Figure 9I,J). Overexpression of *XRCC6* significantly rescued the aforementioned changes induced by H_2_O_2_ treatment (Figure 9G–J).

## 3. Discussion

The dual role of aging in both inhibiting and promoting tumors underscores its significance in cancer biology [12,13]. Cellular senescence is a multifaceted process in which cells irreversibly stop dividing. While it prevents damaged cells from becoming cancerous, senescence can also promote cancer in certain contexts [6]. Throughout tumor progression, factors associated with the senescence-associated secretory phenotype (SASP) can generate an inflammatory milieu that promotes tumor growth [9,14].A previous study developed a risk prediction model for lung adenocarcinoma with six ARGs, demonstrating AUC values of 0.70, 0.62, and 0.65 for 1-, 3-, and 5-year ROC curves in the training set, respectively. Combining this with a nomogram of other clinical factors yielded AUCs of 0.754, 0.730, and 0.742, respectively [15]. A different study developed a risk prediction model for lung adenocarcinoma using 20 senescence-related lncRNAs, achieving AUC values of 0.855, 0.805, and 0.793 for predicting 1-, 3-, and 5-year outcomes, respectively, but this model lacked external dataset validation [16]. Our study developed a prognostic model utilizing 14 ARGs, which demonstrated strong predictive performance in the training set, internal test set, and external validation set. The nomogram incorporating clinical data showed AUCs greater than 0.8 for one year in both datasets.

The risk scores obtained from the model were significantly linked to different facets of the tumor microenvironment, each carrying its own biological significance. Cancer-associated fibroblasts (CAFs) are key constituents of the tumor stroma, facilitating tumor growth and metastasis through the formation of the extracellular matrix and the secretion of cytokines and growth factors [17]. Elevated risk scores derived from our model were positively correlated with the presence of CAFs, suggesting that higher risk scores are linked to a tumor-promoting microenvironment. Additionally, the model’s risk scores were associated with critical elements of immune escape, including T-cell dysfunction and exclusion. T-cell dysfunction is characterized by the reduced ability of T-cells to identify and eliminate cancer cells, typically resulting from prolonged exposure to tumor antigens and the immunosuppressive environment established by the tumor. T-cell exclusion involves physical or functional barriers that prevent T-cell infiltration into tumor tissues [18]. Our findings suggest that elevated risk scores are linked to greater T-cell dysfunction and exclusion, indicating that patients with higher risk scores experience a more pronounced immunosuppressive microenvironment. By correlating the expression of senescence-related genes with these key aspects of the tumor microenvironment, our model underscores the significant role of senescence in cancer biology. This integrated approach emphasizes the model’s effectiveness in predicting prognosis and identifying potential therapeutic targets, offering valuable insights to guide the development of targeted therapies aimed at improving patient outcomes.

XRCC6/Ku70 is an evolutionarily conserved protein that associates with double-strand DNA breaks in the nucleus, facilitating DNA repair via the non-homologous end-joining pathway [19]. Double-strand DNA breaks can significantly jeopardize genomic stability, leading to cellular senescence [20], and improper repair can lead to cancer development [21]. Studies have shown that elevated *XRCC6* expression can confer radiotherapy resistance in tumor cells, including cervical and rectal cancer cells, potentially diminishing the effectiveness of radiotherapy [22,23]. Beyond its well-documented role in DNA repair, multiple studies have highlighted *XRCC6*’s involvement in several additional cellular processes, including apoptosis, senescence, and HIV replication [24]. In this study, we discovered that *XRCC6* is universally upregulated in lung adenocarcinoma, with preliminary bioinformatics analysis indicating its correlation with immune scores and tumor microenvironment scores and enrichment analysis suggesting its association with the cell cycle. Further in vitro experiments showed that knocking down *XRCC6* reduced the proliferative capacity of lung cancer cells, while overexpression of *XRCC6* rescued H2O2-induced DNA damage and cellular senescence, implying a significant biological role of XRCC6 in lung adenocarcinoma.

This study established a prognostic model based on senescence-associated genes, which exhibited strong predictive capabilities in lung adenocarcinoma patients and showed significant correlations with key factors of the tumor microenvironment. *XRCC6* was found to be upregulated in lung adenocarcinoma and associated with the cell cycle, with knockdown of *XRCC6* inhibiting lung cancer cell proliferation. This research provides a novel tool for lung adenocarcinoma prognosis assessment and offers critical insights and directions for the development of personalized therapeutic strategies.

## 4. Method

### 4.1. Datasets

The LUAD data for 572 patients were sourced from The Cancer Genome Atlas (TCGA) database, which includes 59 normal tissues adjacent to cancer, 513 tumor tissues, and corresponding clinical information. The expression profiles and clinical outcomes are publicly available and accessible. To validate the prognosis model based on the TCGA LUAD dataset, another LUAD dataset, namely GSE31210, was obtained from the Gene Expression Omnibus (GEO) as a validation dataset. This dataset includes gene expression data and prognostic information for 226 primary lung adenocarcinoma samples [25]. The five GEO datasets were obtained from the GEO to validate.

The ARGs were obtained from the Aging Atlas database (https://ngdc.cncb.ac.cn/aging/index (accessed on 16 January 2024)).

### 4.2. Prognostic Model Construction and Validation

The chi-square test was conducted to analyze variations in gender, age, tumor stage, invasion depth, lymph node metastasis, distant metastasis, and smoking history across the training, internal testing, and overall datasets.

The univariate Cox regression model was utilized to assess the relationship between continuous ARG expression and overall survival (OS). Hazard ratios (HRs) and *p*-values were utilized to identify potential ARGs associated with survival. An HR greater than 1 indicated risk factors, whereas an HR less than 1 indicated protective factors. ARGs with a *p*-value below 0.05 were deemed survival-related and were incorporated into LASSO and multivariate Cox regression analyses to develop a prognostic model.

Kaplan–Meier plots were employed to compare survival outcomes among different risk groups in each dataset. Additionally, multivariate Cox regression was utilized to evaluate the independence of the risk score from clinicopathological characteristics. The predictive performance of the model was assessed using the area under the time-dependent ROC curve (AUC). The expression and clinical-pathological associations of each ARG in the model were also examined.

### 4.3. Software and Packages

The analyses were performed using R software (Version 4.3.1). A random patient split into 6:4 training and test sets was achieved using “caret” the “care” package. LASSO regression was performed with the “glmnet” package. Univariate and multivariate Cox analyses, along with Kaplan–Meier plots, were generated using “survivalROC” packages and “survminer” packages. The time-ROC curve and AUC were calculated using the “TimeROC” and “survivalROC” packages. Nomograms were created using the “rms” package.

### 4.4. Correlation Analysis

To understand the role of ARGs, correlations were analyzed between the risk score and the expression of tumor suppressor genes (TSGs), Tumor Mutation Burden (TMB), immune regulatory gene expression, immune cell infiltration, and the Tumor Immune Dysfunction and Exclusion (TIDE) score, as well as drug sensitivity score. TSGs and oncogenes were obtained from the TSGene (https://bioinfo.uth.edu/TSGene/ (accessed on 20 January 2024)) [26] database and the ONGene (http://www.ongene.bioinfo-minzhao.org (accessed on 8 February 2024)) [27] database. A total of 11 immune checkpoint genes (ICGs) were extracted from previous studies [28]. Immune cell infiltration scores came from the TIMER2.0 database (http://timer.cistrome.org/ (accessed on 19 February 2024)) [29]. TIDE scores were predicted using the online TIDE tool (http://tide.dfci.harvard.edu/ (accessed on 19 February 2024)) [30]. Data from the Genomics of Drug Sensitivity in Cancer database (GDSC) were utilized to evaluate drug sensitivity with the “oncoPredict” package [31]. Correlations were determined using Spearman’s method from the “psych” package.

### 4.5. Enrichment Analysis

Gene set enrichment analysis (GSEA) was conducted using the “ClusterProfiler” R package to investigate gene set enrichment based on risk score correlations.

Differentially expressed genes (DEGs) between the low- and high-risk groups were identified with the “limma” package, applying criteria of *p*-value < 0.05 and log(fold change) > 1 and analyzed for pathways and biological processes using the DAVID online tool.

### 4.6. XRCC6 shRNA Plasmid Construction

The shRNA sequences for XRCC6 were created utilizing the BLOCK-iT™ RNAi Designer tool (https://rnaidesigner.thermofisher.com/rnaiexpress (accessed on 8 March 2024)). The annealed double-stranded fragments of shRNA were subsequently inserted into the pGreen vector. Following the assessment of knockdown efficiency for several candidate shRNAs, two specific shRNAs targeting XRCC6 were selected for further experiments. Moreover, a scrambled, non-specific control shRNA (shNC) was inserted into the same vector and used as a negative control.

### 4.7. Cell Culture and Transfection

The human lung cancer cell lines A549 and H1299 were obtained from the American Type Culture Collection (ATCC). The Cells were cultured in DMEM supplemented with 10% FBS at 37 °C in a 5% CO_2_ atmosphere. After a 24 h incubation, the lung cancer cells were transfected with 2.5 μg of shRNA using Lipofectamine 6000 reagent (Beyotime, Shanghai, China), following the manufacturer’s protocol.

### 4.8. Cell Proliferation Assay

For the EdU assay, cells were treated with 10μM EdU for 2 h, followed by fixation with 4% paraformaldehyde and permeabilization with 0.3% Triton X-100 in PBS. Click reaction solution from the Beyotime Institute of Biotechnology, China, was used for subsequent processing. After a 24 h incubation, cell images were captured using an inverted fluorescent microscope, and data were analyzed using NIH ImageJ software (Version 1.8.0).

For the CCK-8 assay, cells were incubated with the CCK-8 working solution for 1–4 h at 37 °C. Following this, the absorbance was measured at a wavelength of 450 nm using a microplate reader. The obtained absorbance values were then used to calculate cell viability percentages relative to control samples.

### 4.9. Transwell Migration Assay

Cells from each experimental group were placed in the upper chambers of Transwell membranes (Corning, Inc., Corning, NY, USA). The upper chamber contained medium without fetal bovine serum (FBS), while the lower chamber contained complete medium. After a 24 h incubation, the cells were fixed in methanol, stained with 0.5% crystal violet, and visualized under a microscope. Cell migration was quantified using NIH ImageJ software (Version 1.8.0) following washing with phosphate-buffered saline (PBS, Gibco, Grand Island, NY, USA).

### 4.10. Quantitative Polymerase Chain Reaction (qPCR) Assay

Total RNA was isolated from cultured cells using a commercial RNA isolation kit in accordance with the manufacturer’s instructions. The RNA concentration and purity were evaluated using a spectrophotometer. Following this, cDNA synthesis was carried out using a reverse transcription kit. Quantitative PCR (qPCR) was performed on a real-time PCR system with specific primers targeting *CDKN1A*, *CDKN2A*, and a reference gene. The relative expression levels of *CDKN1A* and *CDKN2A* mRNA were assessed using the comparative Ct method.

### 4.11. Detection of Cellular Senescence

Senescent cells were detected using a fluorescein-based probe targeting β-galactosidase activity, a widely used marker for senescence. The probe, containing two galactoside moieties, is cleaved by β-galactosidase within lysosomes under acidic conditions, emitting a fluorescent signal with absorption/emission maxima of 490/514 nm. Following incubation with the probe, cells were fixed, and nuclei were counterstained with DAPI. Fluorescent signals indicative of senescent cells were visualized using a fluorescence microscope equipped with standard filter sets.

### 4.12. Immunofluorescence

Cells were cultured on glass coverslips in 6-well plates and exposed to various treatments. After treatment, cells were fixed with 4% paraformaldehyde for Z minutes, permeabilized with 0.3% Triton X-100 in PBS for A minutes, and blocked with 5% bovine serum albumin (BSA) for B minutes. Subsequently, cells were incubated with primary anti-γH2AX antibody diluted in blocking buffer overnight at 4 °C. After washing, cells were incubated with a fluorescently labeled secondary antibody for C hours at room temperature. Nuclei were counterstained with DAPI, and coverslips were mounted onto glass slides using a fluorescent mounting medium. The fluorescence signals indicating DNA damage were visualized and captured using a fluorescence microscope.

### 4.13. Statistical Analyses

Statistical analyses were conducted with GraphPad V8.3.0 software (GraphPad Software, LLC, La Jolla, CA, USA). The data were presented as mean ± standard deviation. Statistically significant differences between groups were assessed using Student’s *t*-test for two-group comparisons and analysis of variance (ANOVA) for comparisons involving multiple groups. All statistical tests were two-tailed, and a *p*-value < 0.05 was considered statistically significant.

## Figures and Tables

**Table 1:** Demographic and clinical data for the training and internal testing sets.

Characteristics		TCGA_LUAD			Chi-Square *p*-Value
		Training (*n* = 303)	Internal Testing (*n* = 201)	All	
Gender	female	159 (52.48%)	111 (55.22%)	270 (53.57%)	0.832
	male	144 (47.52%)	90 (44.78%)	234 (46.43%)	
Age	≤60	95 (32.09%)	63 (31.82%)	158 (31.98%)	0.998
	>60	201 (67.91%)	135 (68.18%)	336 (68.02%)	
M	M0	202 (93.09%)	133 (93.01%)	335 (93.06%)	1.000
	M1	15 (6.91%)	10 (6.99%)	25 (6.94%)	
N	N0	187 (64.04%)	137 (69.19%)	324 (66.12%)	0.497
	N1/2	105 (35.96%)	61 (30.81%)	166 (33.88%)	
T	T1/2	260 (85.81%)	178 (88.56%)	438 (86.90%)	0.670
	T3/4	43 (14.19%)	23 (11.44%)	66 (13.10%)	
Stage	Stage I/II	230 (75.91%)	160 (79.60%)	390 (77.38%)	0.624
	Stage III/IV	73 (24.09%)	41 (20.40%)	114 (22.62%)	
Smoke history	Non-smoke	129 (42.57%)	71 (35.32%)	200 (39.68%)	0.265
	Smoke	174 (57.43%)	130 (64.68%)	304 (60.32%)	
time	≤2	174 (57.43%)	111 (55.22%)	285 (56.55%)	0.888
	>2	129 (42.57%)	90 (44.78%)	219 (43.45%)	
status	Live	189 (62.38%)	132 (65.67%)	321 (63.69%)	0.753
	Dead	114 (37.62%)	69 (34.33%)	183 (36.31%)	

## Data Availability

The raw data supporting the conclusions of this article will be made available by the authors on request.

## Supplementary Materials

The following supporting information can be downloaded at: https://www.mdpi.com/article/10.3390/ijms252413572/s1.

## Abbreviations

ARGs	Aging-related genes	TMB	Tumor Mutation Burden
LUAD	Lung adenocarcinoma	TIDE	Tumor Immune Dysfunction and Exclusion
TCGA	The Cancer Genome Atlas	TSG	Tumor suppressor gene
LASSO	Least Absolute Shrinkage and Selection Operator	ICGs	Immune checkpoint genes
AUC	Area Under the Curve	HRs	Hazard ratios
CAFs	Cancer-associated fibroblasts	OS	overall survival
WHO	The World Health Organization	GDSC	The Genomics of Drug Sensitivity in Cancer Database
NSCLC	Non-small cell lung cancer	DEGs	Differentially expressed genes
SCLC	Small cell lung cancer	GSEA	Gene set enrichment analysis
LUSC	Lung squamous cell carcinoma	MAPK	Mitogen-activated protein kinase
LCC	Large cell carcinoma	GSEA	Gene Set Enrichment Analysis
GEO	Gene Expression Omnibus	CAF	Cancer-associated fibroblasts
ROC	Receiver Operating Characteristic Curve	KEGG	Kyoto Encyclopedia of Genes and Genomes
TAM	Tumor-associated macrophages	MDSC	Myeloid-derived suppressor cell
DCA	Decision Curve Analysis	LncRNA	Long non-coding RNA
