# Supplementary material for: Aging-Related Gene-Based Prognostic Model for Lung Adenocarcinoma: Insights into Tumor Microenvironment and Therapeutic Implications
Source: Int J Mol Sci. 2024 Dec 18;25(24):13572. doi: 10.3390/ijms252413572 (PMC11678022; doi:10.3390/ijms252413572)
Supplement: Supplementary file 1 [file ijms-25-13572-s001.zip › ijms-3310717-supplementary.pdf]

# Table·S1

| Characteristics | | All |
|---|---|---|
| Stage | Stage I | 168 (74.34%) |
| | Stage II/III | 58 (25.66%) |
| MYC_copy | >1 | 169 (74.78%) |
| | ≤1 | 57 (25.22%) |
| Smoke_history | Ever-smoker | 111 (49.12%) |
| | Never-smoker | 115 (50.88%) |
| Age | ≤60 | 108 (47.79%) |
| | >60 | 118 (52.21%) |
| Gender | Male | 105 (46.46%) |
| | Female | 121 (53.54%) |
| time | ≤5 | 124 (54.87%) |
| | >5 | 102 (45.13%) |
| status | Live | 191 (84.51%) |
| | Dead | 35 (15.49%) |

**Table S1.** Demographic and clinical data for conceptual data

# Figure·S1

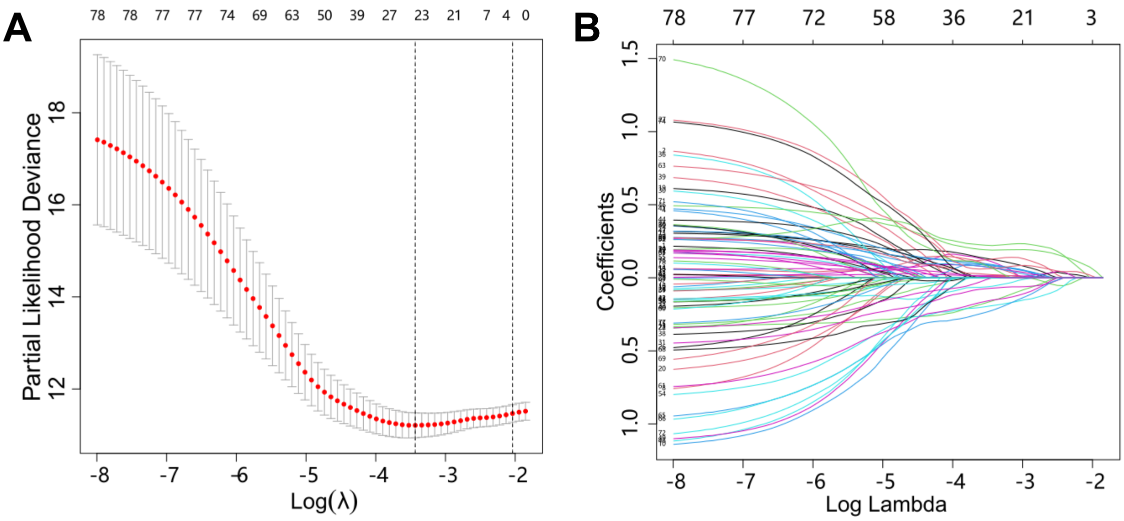


**Figure S1.** Variable Screening Based on LASSO Regression.(A)The selection process of the optimum value of the parameter λ in the Lasso regression model by cross-validation method. (B)The variation characteristics of the coefficient of variables.

# Figure·S2

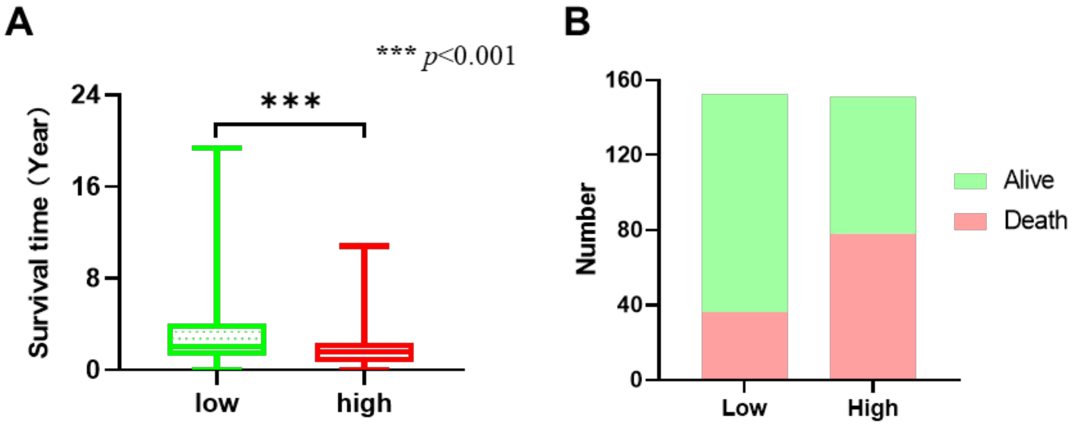


**Figure S2.** (A) Analysis on the difference of total survival time between patients with high risk score and patients with low risk score in training queue.(B)Death of patients with high risk score and patients with low risk score in training queue.

# Figure·S3

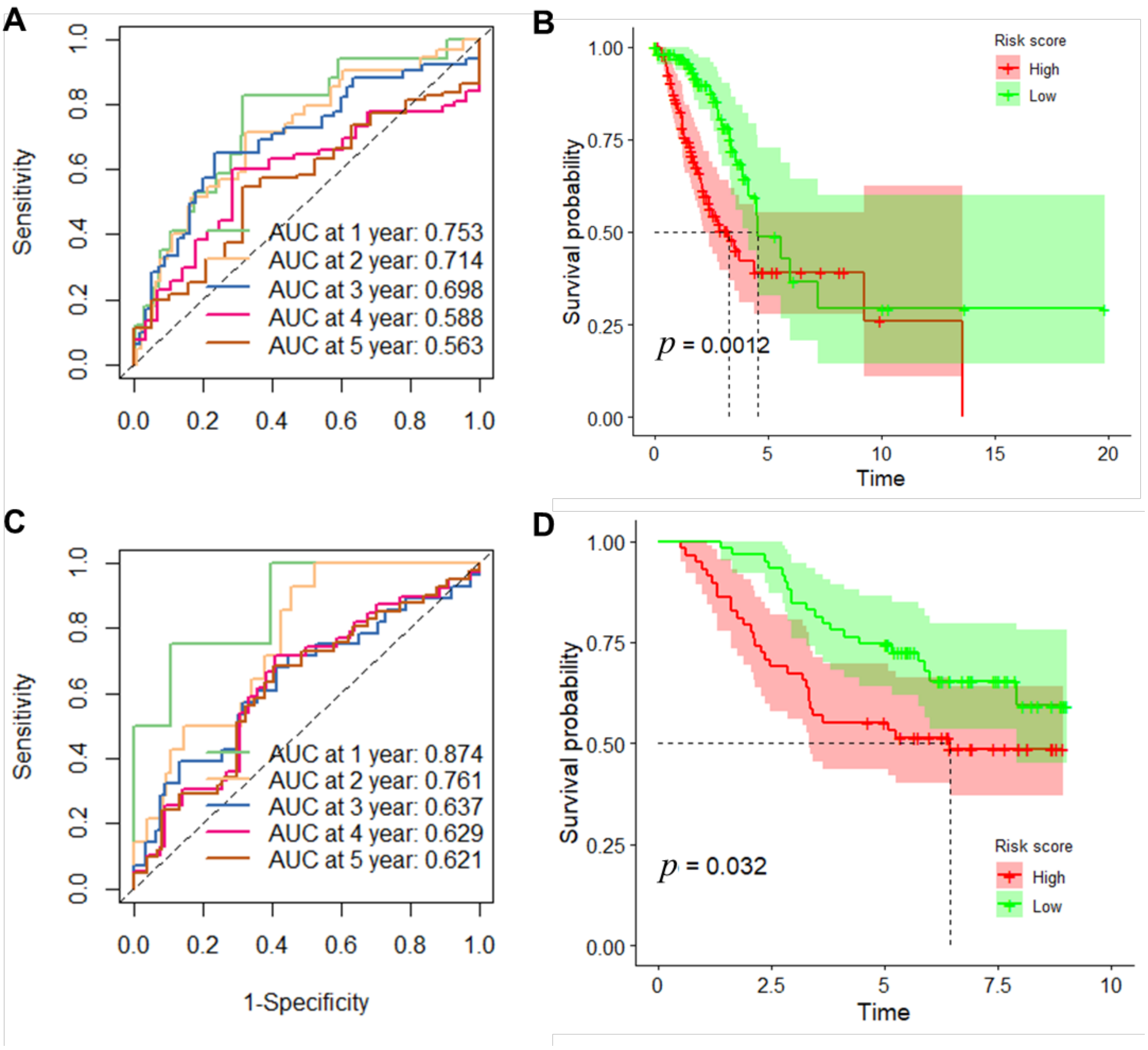


**Figure S3.** (A)Receiver operating characteristic (ROC) of the internal test cohort/Diagnostic outcomes are shown via receiver operating characteristic (ROC) curves for the performance of model . /TimeROC curves displaying ROC curves and AUC values for 1-5 years in the internal test cohort (B) Kaplan-Meier survival curve of risk score/Survival curves for high- and low-risk groups in the internal test cohort

# Figure·S4

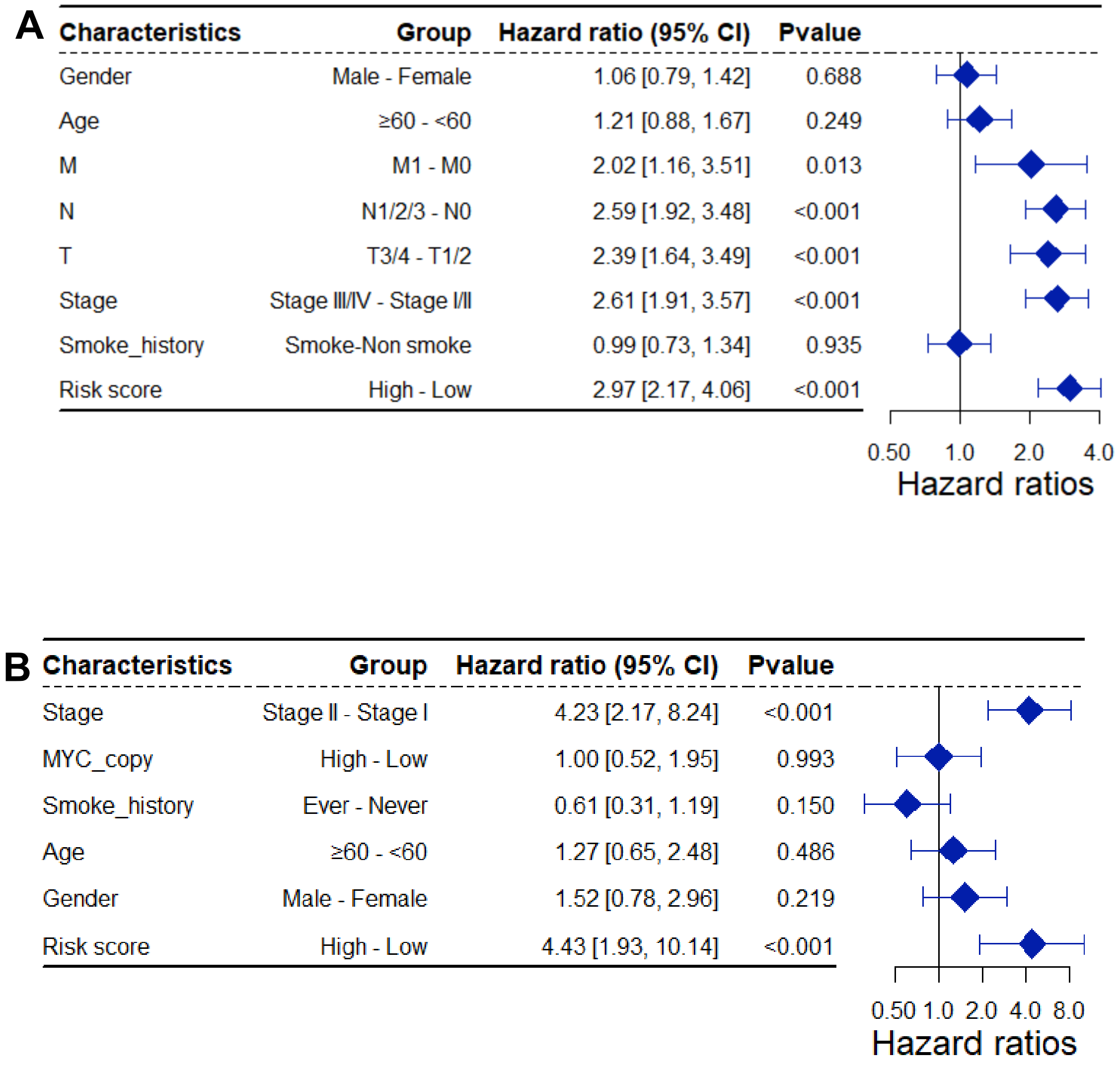


**Figure S4.** (A)Results of multivariate COX analysis incorporating significant factors from univariate COX analysis in the TCGA_LUAD dataset.(B)Results of multivariate COX analysis of the GSE31210 dataset.

# Figure·S5

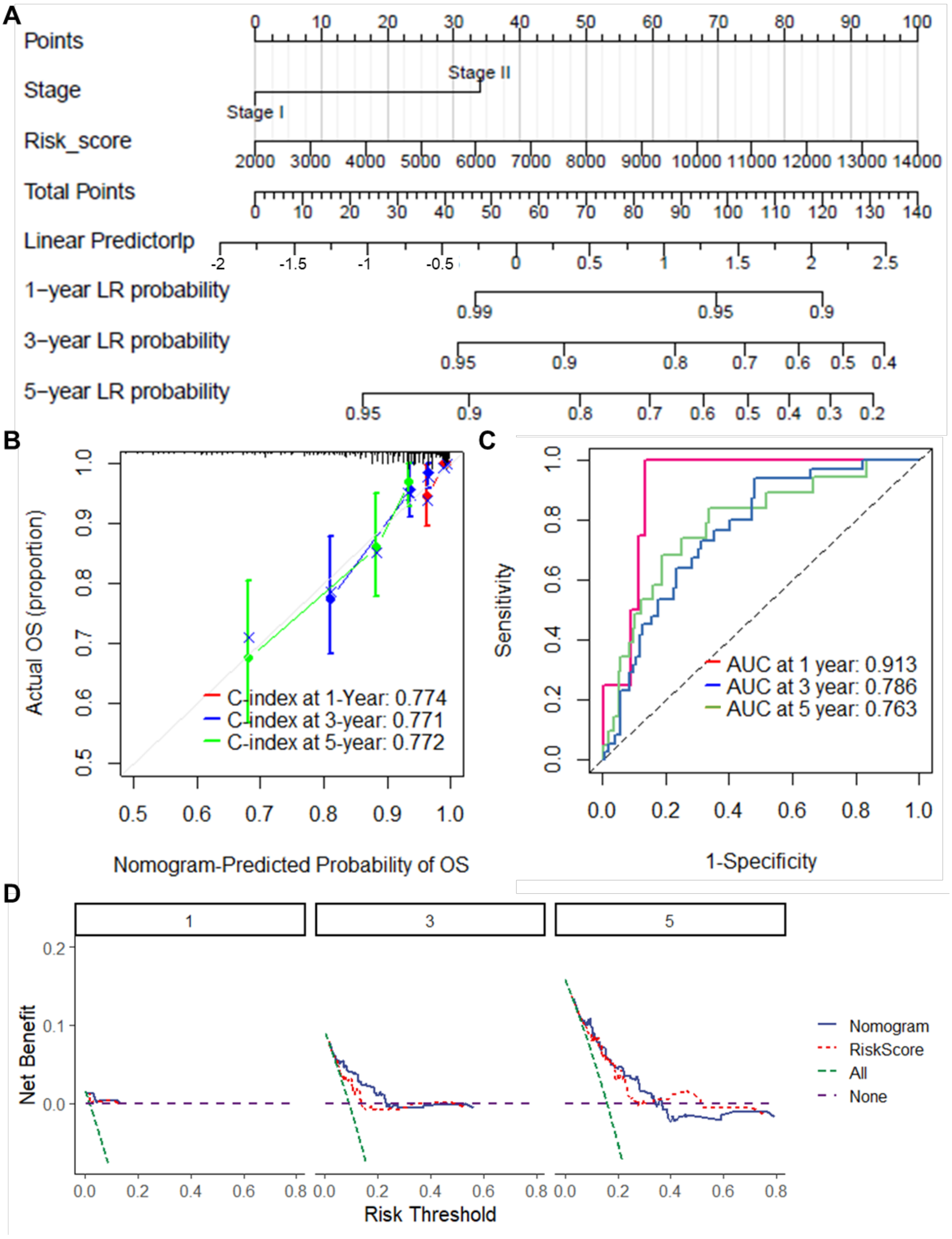
A
Points
Stage
Stage II
Stage I
Risk_score
Total Points
Linear Predictorlp
1−year LR probability
3−year LR probability
5−year LR probability
B
Actual OS (proportion)
Nomogram-Predicted Probability of OS
C-index at 1-Year: 0.774
C-index at 3-year: 0.771
C-index at 5-year: 0.772
C
Sensitivity
1-Specificity
AUC at 1 year: 0.913
AUC at 3 year: 0.786
AUC at 5 year: 0.763
D
Net Benefit
Risk Threshold
Nomogram
RiskScore
All
None

**Figure S5**. Construction and validation of the clinical predictive nomogram based on the GSE31210 dataset. (A)Clinical predictive nomogram incorporating independent prognostic factors identified through multivariate COX regression analysis, with the total points projected on the bottom scale representing the probabilities of 1-, 3-, and 5-year overall survival; (B) Calibration plots for the 1-, 3-, and 5-year overall survival predictions based on the nomogram in the TCGA_LUAD dataset; (C) Kaplan–Meier curves for overall survival based on nomogram scores in the TCGA_LUAD dataset; (D) DCA curves for the nomogram and risk score predicting 1-, 3-, and 5-year OS in the TCGA_LUAD dataset. ARGs, aging-related genes; OS, overall survival; DCA, decision curve analysis.

Figure·S6

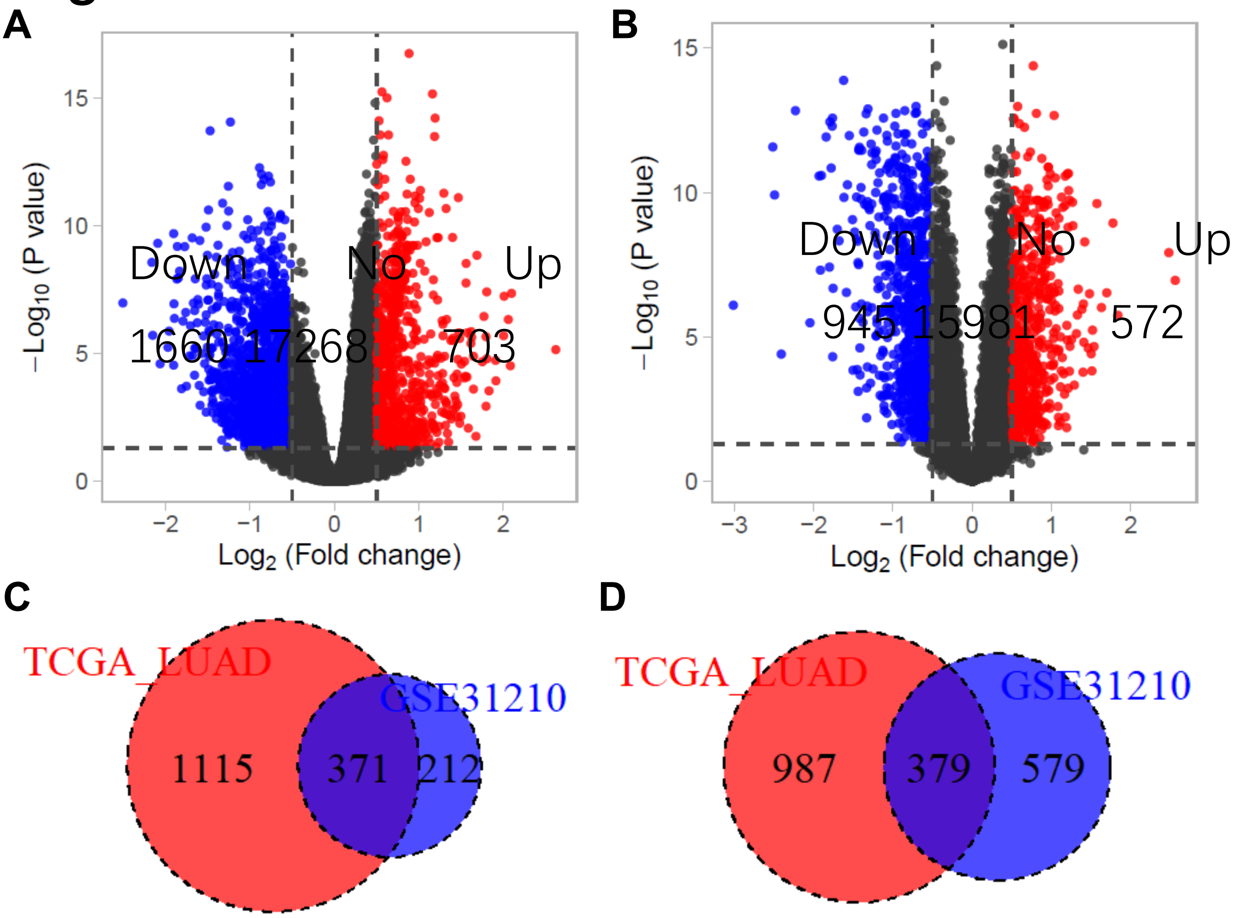


**Figure S6. (A)** Differential expression analysis between high-risk group and low-risk group in TCGA-LUAD data set. (B) Differential expression analysis between high-risk group and low-risk group in GSE31210 data set. (C) Genes upregulated in TCGA-LUAD and GSE31320. (D) Genes down-regulated in TCGA-LUAD and GSE31320.

# Figure·S7

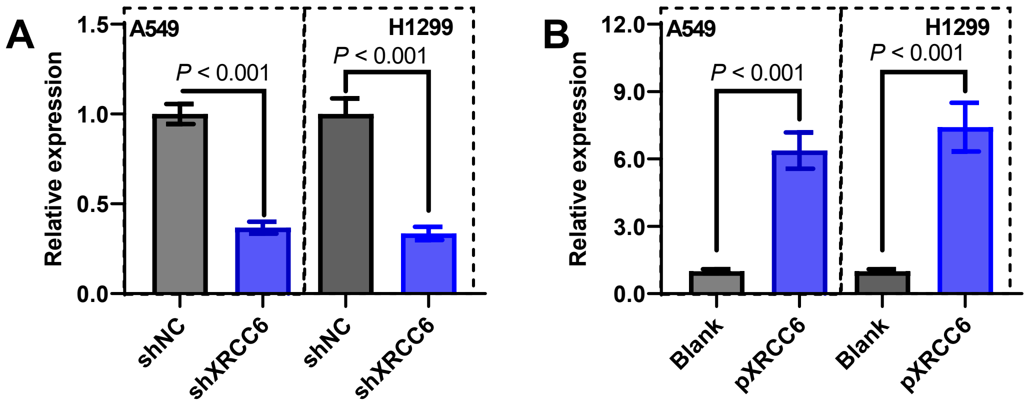


**Figure S6. (A)** QPCR was used to detect the level of XRCC6 in H1299 and A549 cells after XRCC6 was knocked down. (B) QPCR detected the level of XRCC6 in H1299 and A549 cells after overexpression of XRCC6.
